# Patient with ovarian insufficiency: baby born after anticancer therapy and re-transplantation of cryopreserved ovarian tissue

**DOI:** 10.1186/s13048-020-00713-9

**Published:** 2020-09-29

**Authors:** Vladimir Isachenko, Bernd Morgenstern, Plamen Todorov, Evgenia Isachenko, Peter Mallmann, Bettina Hanstein, Gohar Rahimi

**Affiliations:** 1 Department of Obstetrics and Genecology, University Maternal Hospital, Research Group for Reproductive Medicine and IVF-Laboratory, Cologne University, Cologne, Germany; 2 Institute of Biology and Immunology of Reproduction, Sofia, Bulgaria

**Keywords:** Baby born, Cancer, Cryopreservation, Cryoprotectants, Human ovarian tissue, Re-transplantation

## Abstract

**Background:**

The second major cause of death is cancer. In fact, the effectiveness of anticancer treatments and positive long-term prognosis for young women has increased. However, the problem of post-cancer infertility plays a significant role, because chemotherapy can be gonadotoxic and lead to the functional death of ovaries. There is potential key solution to this problem: cryopreservation of ovarian tissue before cancer therapy with re-implantation after convalescence. Data regarding cryopreservation and re-transplantation of ovarian tissue from patients with ovarian insufficiency is limited. The aim of this treatment was the re-transplantation of cryopreserved ovarian tissue after anticancer therapy of patient with ovarian insufficiency (56 IU/l FSH, 8 ng/l β-estradiol, < 1.1 ng/ml anti-Mullerian hormone, 1 primary follicle per 10mm^3^).

**Case presentation:**

After the operation, four tissue fragments (10–16 × 8–13 × 1.0–1.2 mm) were cooled to 5 °C in the freezing medium (culture medium+ 6% ethylene glycol+ 6% dimethyl sulfoxide+ 0.15 M sucrose) for 24 h, frozen and thawed. Freezing was performed in four standard 5 ml cryo-vials with ice formation at − 9 °C, cooling from − 9 to − 34 °C at a rate of − 0.3 °C/min and plunging at − 34 °C into liquid nitrogen. After thawing in a 100 °C (boiling) water bath, the removal of cryoprotectants was performed in 0.5 M sucrose with 20 min. exposure in sucrose and 30 min. stepping rehydration. After thawing of one cryo-vial, part (5 mm^3^) of experimental ovarian tissue after 7 day in vitro culture was histological evaluated and two ovarian fragments (8 × 7 × 1.0 mm and 7 × 6 × 1.0 mm) were re-transplanted. The quantity of follicles after cryopreservation and in vitro culture was not increased (*P* > 0.1): it was found 1 primordial follicle in 5 mm^3^ of tissue. Thirty seven days after the re-transplantation of ovarian tissue, the restoration of the menstrual cycle of Patient W. was noted. Three months after the transplantation, the patient became spontaneously pregnant and delivered a healthy baby girl at term.

**Conclusions:**

Described protocol of conventional cryopreservation of ovarian tissue can be used for treatment of patients with ovarian insufficiency.

## Background

The second major cause of death is cancer [1]. In fact, the effectiveness of anticancer treatments and positive long-term prognosis for young women has increased. However, the problem of post-cancer infertility plays a significant role, because chemotherapy can be gonadotoxic and lead to the functional death of ovaries. There is potential key solution to this problem: cryopreservation of ovarian tissue before cancer therapy with re-implantation after convalescence [2]. Now this procedure is a routine: more than 130 live births from cryopreserved ovarian tissue have been reported worldwide as of June 2017 [3]. However, the data regarding cryopreservation and re-transplantation of ovarian tissue of patients with ovarian insufficiency is limited**.**

The aim of this treatment was the re-transplantation of cryopreserved ovarian tissue after anticancer therapy of patient with ovarian insufficiency (56 IU/l FSH, 8 ng/l β-estradiol, < 1.1 ng/ml anti-Müllerian hormone, 1 primary follicle per 10mm^3^).

## Case presentation

This study was approved by the Ethics Boards of University Cologne (applications 99,184 and 13–147). Written informed consents were obtained from participant involved in our study.

Except where otherwise stated, all chemicals were obtained from Sigma (Sigma Chemical Co., St. Louis, MO, USA).

### Design of investigations

Two series of investigations were carried out. First series of experiments included thawing and re-transplantation of ovarian tissue. Experiments of second series were performed on the day of thawing and re-transplantation. It was evaluated the quantity of follicles in thawed ovarian tissue.

### Tissue collection, dissection, pre-cooling, cryopreservation (freezing and thawing) and in vitro culture

The medium used for transport and dissection, the culture (basal) medium, was comprised of Leibovitz L-15 with 5% Dextran Serum Substitute (Irvine Sci., Santa Ana, CA, USA). After removal of whole left ovary, this ovary was kept at a temperature of 32 to 34 °C, and then was transported to the laboratory within 10 min of surgery. Using tweezers and a No. 22 scalpel, the ovary was dissected into four medulla-contained fragments. Parallel with cryopreservation of four large fragments for following clinical transplantation, 10 small pieces (1.0 × 1.0 × 1.0 mm) were been frozen for following evaluation of follicles after thawing. In accordance with SOP protocol of our clinic, on the day of operation 10 mm^3^ of fresh ovarian tissue was evaluated for detection of follicles. Then, the large fragments and experimental pieces were cooled at 5 °C for 24 h in the freezing medium (basal medium + 6% (v/v) ethylene glycol + 6% (v/v) dimethyl sulfoxide + 0.15 M sucrose). Four ovarian fragments and 10 small pieces were frozen the next day as described below.

The procedure of freezing and thawing was performed as published previously [4–10]. Pre-cooling of the ovarian tissue was performed in the presence of cryoprotectants (in the freezing medium) as described earlier [11]. The freezing medium composed of the culture medium supplemented with freezing solution. Freezing was performed in standard 5 ml cryo-vials with ice formation at − 9 °C, cooling from − 9 to − 34 °C at a rate of − 0.3 °C/min and plunging at − 34 °C into liquid nitrogen. Sixteen seconds thawing was performed in a 100 °C (boiling) water bath as described earlier [6, 8, 12]. The removal of cryoprotectants was performed in 0.5 M sucrose [8] with 20 min. exposure in sucrose and 30 min. stepping rehydration. The last step involved three washes in basal medium for 10 min. immediately prior to placement for in vitro culture or for transplantation into the patient.

In vitro culture of tissue was performed as described earlier [6], with some modifications. Ovarian tissue pieces were transferred to 700 ml dishes for suspension culture (Cellstar™, Greiner Bio-One GmbH) in 100 ml AIM-V™ medium for tissue culture (Gibco, Grand Island, NY, USA) and cultured for 7 days in vitro in air at 37 °C in 5% CO_2_, with agitation at 75 osc/min using a rotating orbital shaker (N-Biotek, Bucheon, Korea). Five ovarian pieces were cultured in one culture dish. After the culture, histological evaluation of tissue was performed. Examples of normality of follicles and of the different follicular degenerations can be observed elsewhere [7, 9].

Experiments of second series were performed on the day of thawing and re-transplantation of ovarian tissue. It was evaluated the quantity and quality of follicles in frozen and thawed ovarian tissue.

### Patient W

**Fig. 1:**
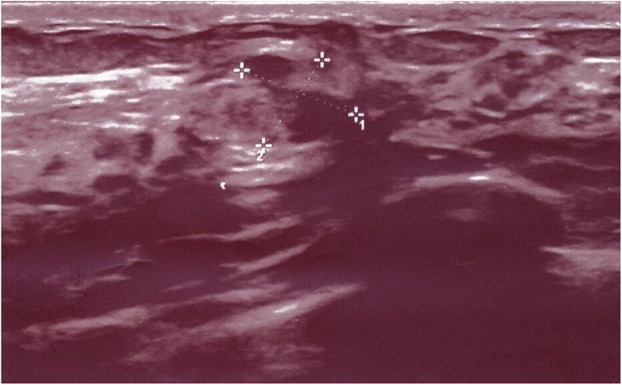
Patient W.: Mamma-Ca was diagnosed in 2015

Patient W. was born in 1981 and, in 2015 (Patient W. was 33 y. o.), she developed an invasive ductal Mamma-Ca pT1b pN0 (073 sn) M0 V0 L0 G2 Pn0 R0, 4 × 9 mm (Fig. 1). The tumour was resected. Chemotherapy (3 weeks primary anti-hormonal therapy with Tamoxifen, 8 cycles Nab-Paclitaxel and 4 cycles Epirubicin/ Cyclophosphamide) was administered. Before beginning of chemotherapy it was detected the pre-menopausal status. Before cryopreservation of ovarian tissue (Patient W. was 33 y. o., had body length 165 cm and weight 58 kg, BMI =22) and beginning of anticancer treatment it was detected the idiopathic ovarian insufficiency (low ovarian reserve of unknown cause) of Patient W.: 56 IU/l FSH, 8 ng/l β-estradiol, < 1.1 ng/ml anti-Müllerian hormone.

**Fig. 2:**
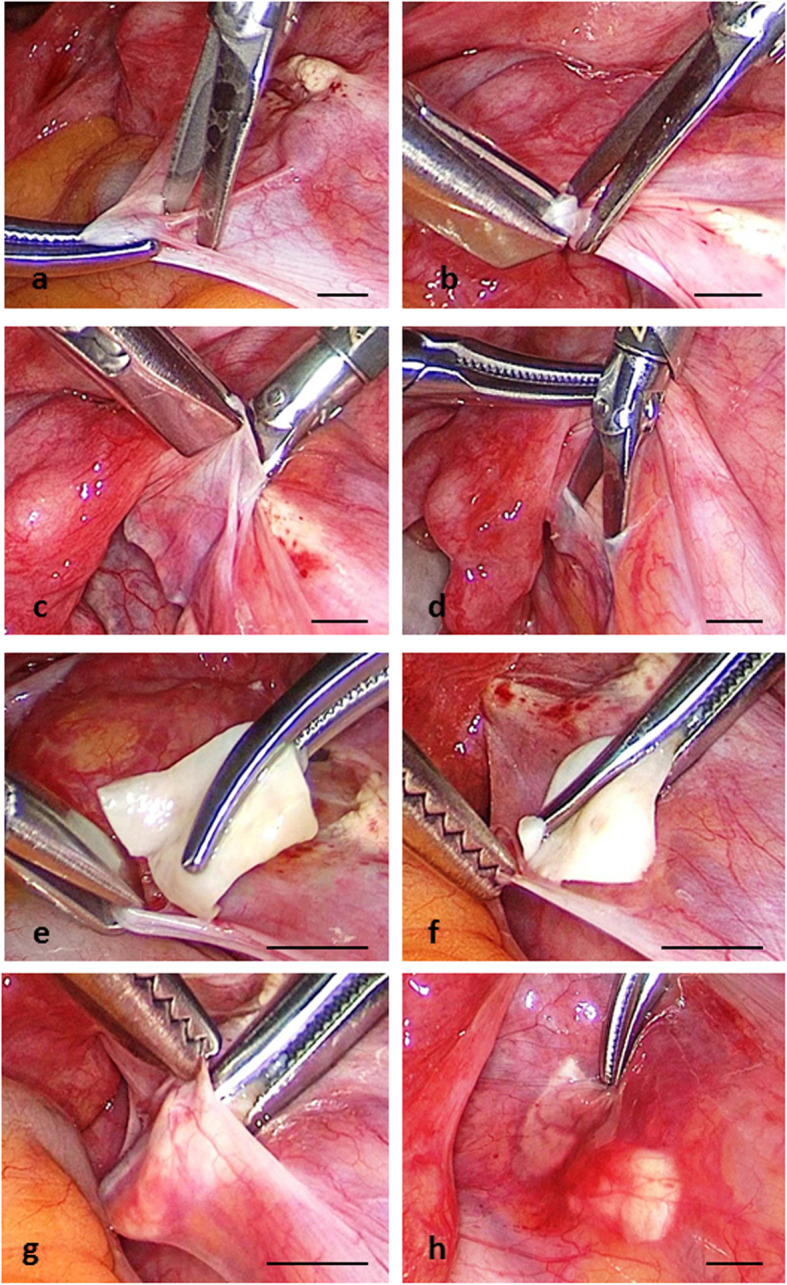
Transplantation of frozen ovarian tissue of Patient W. in 2019. **a**, **b**, **c**, **d** Formation of two separated peritoneal windows. **e**, **f**, **g** Insertion of two thawed ovarian fragments into two peritoneal windows. **h** Two ovarian fragments in two separated peritoneal windows on then end of transplantation. Bar = 5.0 mm

From Patient W. it was obtained informed consent. Freezing of the ovarian tissue of Patient W. was performed as described above. Ovarian tissue was removed from the left ovary, and the right ovary was intact. After partial removal of the medulla, it was formed four ovarian fragments (10–16 × 8–13 × 1.0–1.2 mm) and these fragments were frozen (4 cryo-vials for 4 fragments and 2 cryo-vials for 10 experimental pieces). In 2019, 4 years after the end of anticancer treatment (Patient W. was 37 y. o.), 25% of the ovarian tissue (1 of 4 cryo-vials) was thawed. Ovarian fragment was cut into two parts (8 × 7 × 1.0 mm and 7 × 6 × 1.0 mm) and re-transplantated in the peritoneal window on the left side (Fig. 2).

Thirty seven days after the re-transplantation of ovarian tissue, the restoration of the menstrual cycle of Patient W. was noted.

After 7 days of in vitro culture, the experimental ovarian pieces were observed to have developed a spherical shape. It was histologically evaluated 5 mm^3^ from 10mm^3^ of tissue. It was found one primary follicle of good quality.

**Fig. 3:**
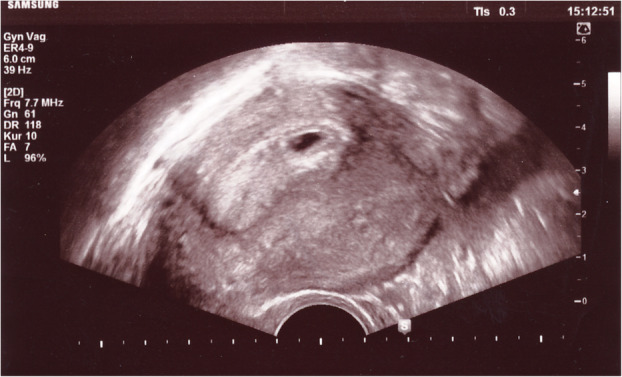
Spontaneous pregnancy of Patient W. 3 months after re-transplantation of ovarian tissue: ultrasound showing 3 week pregnancy

Three months after the autotransplantation Patient W. became spontaneously pregnant (Fig. 3) and later delivered a healthy baby girl (3370 g) at term.

## Discussion and conclusion

**Fig. 4:**
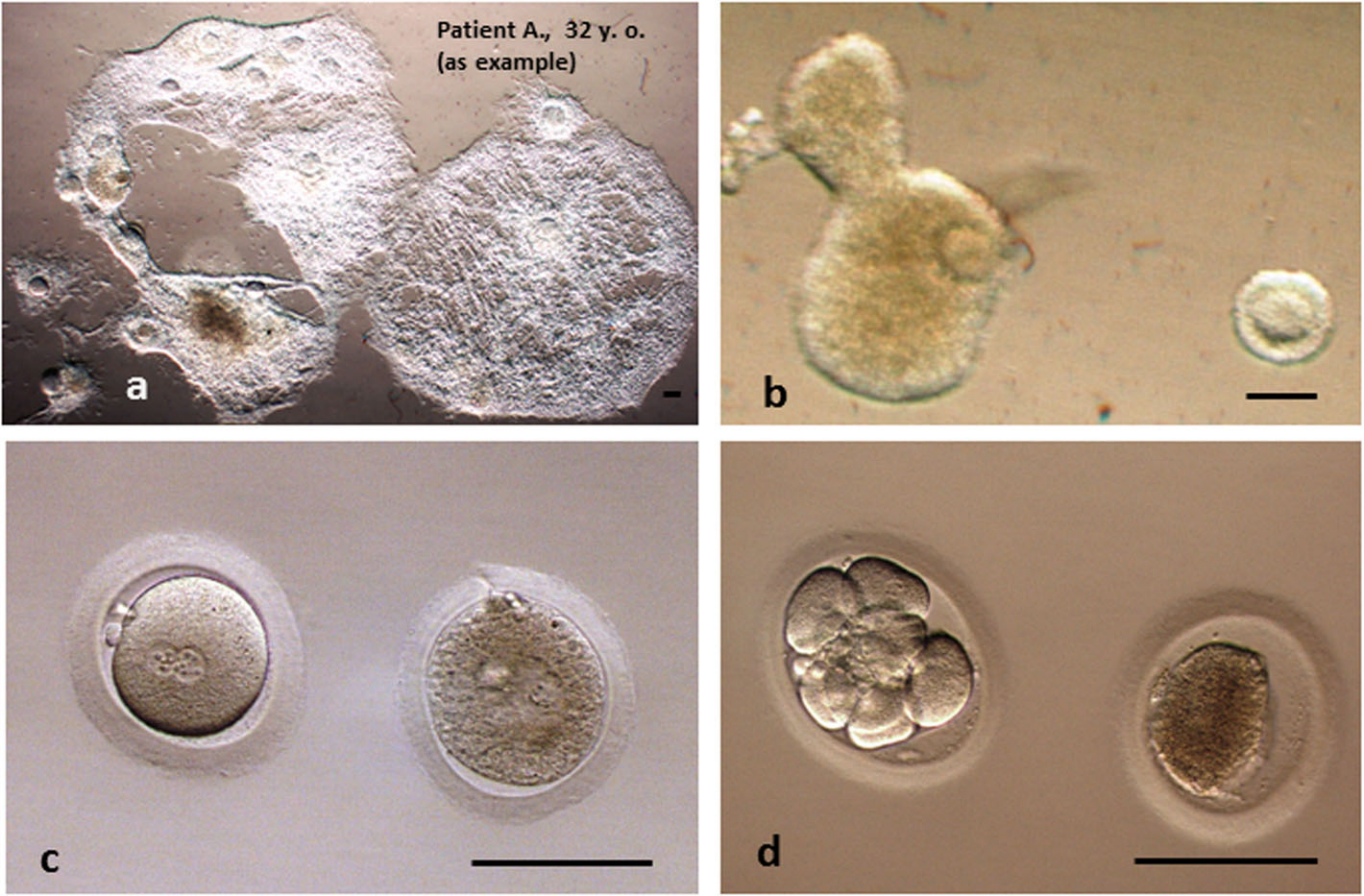
Embryos of Patient W. obtained after preparation of ovarian fragments. **a** Comparative example of a pool of GV-oocytes obtained after preparation of ovarian tissue from another patient after in vitro maturation to M-II stage (29 h). **b** GV-oocytes before in vitro maturation to M-II stage (28 h) and cryopreservation (vitrification). **c** PN-embryos after warming of M-II oocytes and ICSI. **d** Three-days normal embryo and degenerated oocyte before transfer

In accordance with concept of our cryobank, prior to the freezing of ovarian tissue, immature follicles should be punctured to obtain GV-oocytes. Also in the process of preparation of four fragments (cutting of tissue and partial removal of medulla, we obtain spontaneously a number of GV-oocytes. Cryopreservation of both ovarian cortex and oocytes offers the possibility of “double-fertility preservation” [12]. Just after the end of fragments preparation, we check the bottom of Petri dish, collect GV-oocytes (oocytes cumulus complexes) for following maturation to MII stage and cryopreservation (vitrification). From patient it is possible to obtain as many as 20 good quality GV-oocytes (Fig. 4a as example). After the respective manipulations with ovarian tissue of Patient W., we have found two GV-oocytes. These oocytes were matured to MII stage, cryopreserved and fertilized after warming. One normal and one degenerated derived embryos (all in vitro cultured oocytes must be transferred independently from the developmental stage) were transplanted (Fig. 4 b, c, d). No implantation was noted. The aim of mentioned above is additionally to show the low number of obtained GV-oocytes that can be explained by the low ovarian reserve of Patient W.

It is known that primordial follicles in premature ovarian failure (POF) patients cannot be activated normally, and mature oocytes cannot be obtained for in vitro fertilization [13]. However, in vitro activation (IVA) of primordial follicles, allows them to grow to a developed stages [3, 14]. IVA technology offers a new potential treatment for such patients [14–16].

IVA procedure exists in form of drug-included [17–19] as well as drug-free [20–24] treatments.

Drug-free activation can be realized in different forms, such as scraping [24], biopsy [21, 23], sectioning [22, 23]. Hippo signaling pathway is an important intracellular signaling pathway that plays an important role in controlling cell proliferation and determining tissue size, and it is conserved in all multicellular animals [25]. All drug-free treatments include destructive manipulations with ovarian tissue and probably include the interruption of ovarian Hippo signaling expenses.

It has been reported that fragmentation of ovaries induced a transient increase in the polymerization of G-actin to F-actin, decreases in phospho-YAP levels, and increases in nuclear localization of YAP [26]. Our protocol of preparation of ovarian fragments from Patient W. includes scarification and shredding of tissue. By opinion of Kawamura et al. [26], shredded ovarian tissue may be available for Hippo signal activation in our case.

We offer the following hypothesis which can explain the effectivity of retransplantation of ovarian tissue of Patient W. with very low ovarian reserve. Fact is that at least five negative effects observed during cells cryopreservation: hypoxia, increasing of intracellular Ca^2+^, osmotic disruption of cellular membranes, generation of reactive oxygen species (ROS) and lipid peroxidation. Each from these factors can lead to the interruption of ovarian Hippo signaling expenses. By this fact can be explain a positive effect of cryopreservation on the following development of follicles. In that case, we offer to evaluate the cryopreservation procedure of ovarian tissue as in vitro activation of follicles. Certainly, the aim of cryopreservation of ovarian tissue is storage of cells function after thawing. For this purpose we offer the cryopreservation technology described here.

In conclusion, described protocol of conventional cryopreservation of ovarian tissue can be used for treatment of patients with ovarian insufficiency.

## Data Availability

Full availability of data and material are declared. Also the datasets used and/or analyzed during the current study are available from the corresponding author on reasonable request.

## Abbreviations

IU: international units
FSH: follicle stimulations hormone
LH: luteinizing hormone
BMI: Body mass index
YAP: Yes-associated protein
